# Supplementary material for: Learning curve and surgical outcome of robotic assisted colorectal surgery with ERAS program
Source: Sci Rep. 2022 Nov 29;12:20566. doi: 10.1038/s41598-022-24665-w (PMC9709162; doi:10.1038/s41598-022-24665-w)
Supplement: Supplementary file 1 — Supplementary Information 1. [file 41598_2022_24665_MOESM1_ESM.zip › LSC Raw data-Ñ[▒K/133/O_ñw╝╨░O▒Kñσ.pdf]

正式摘要(電子簽章：0000002238199941)

W83 / W83

臺中榮民總醫院

Operation Note

[redacted]

- 手術日期：2021/08/19
- 手術醫師：VS 蔣鋒帆，VS 黃偘霖，R 洪浚嚴，R 郭連雄
- Anesthesia : GE
- Position : Supine
- Aseptic agents : Betadine Aqua

Diagnosis

- Pre-operative diagnosis : A COLON CA cT3N2M0
- Post-operative diagnosis : Ditto.

Surgical Indication

- Primary tumor resection

Operation

- 1. Laparoscopic right hemicolectomy with side to side anastomosis (OpenGIA 80-3.8 X2 were applied)
  2. Ileo-colic artery, right colic artery and middle colic, right branch artery were all ligated via Hemo-clips.
  3. Take down of hepatic flexure : (+)
  4. Cut margin: proximal (ileum) 12 cm, (colon) nearly 1.5 cm, distal 6 cm.
  5. D3 lymph node dissection to the root of right colic artery (station 213)
  6. Repair of mesentery defect : Nil.
  7. ADHESION BARRIER FILM was placed below the central abdominal wound.

Finding

- 1. A 5 X 4 cm tumor over A-colon area
  2. No other gross tumor was found.
  3. There was no visible liver metastasis or visible peritoneal seeding.

Procedure

- 1. General endotracheal anesthesia was induced and then the patient was placed in the supine position.
  2. Preoperative antibiotics were given. A Foley catheter and nasogastric tube were placed. The abdomen was prepped and draped in standard sterile fashion.
  3. A small vertical incision was made left to the umbilicus and a 12mm trocar was inserted into the peritoneal cavity by open method.
  4. Pneumoperitoneum was established with CO2 inflation. Laparoscope was inserted and then a 12mm trocar were inserted over the central wound of abdomen.
  5. Another 5mm trocar was inserted over upper midline of abdomen ; The other 5mm trochars were inserted over RUQ of abdomen and RLQ of abdomen, respectively.
  6. The small bowel was inspected and retracted to downward by grasper. The peritoneum overlying the mesentery was then scored with electrocautery and the ileocolic artery was identified, ligated with endoclips, and transected via Harmonic scalpel.
  7. The remaining vessels to the cecum were also divided and ligated. Using electrocautery and Harmonic scalpel, omentum was freed from the transverse colon and lesser sac was entered.
  8. The middle colic, right branch artery and right colic artery were all further ligated via Hemo-clips and Harmonic scalpel.
  9. After the vessel were all under controlled, the colon was freed from its peritoneal attachments

- along the line of Toldt from the cecum to the hepatic flexure.
  10. Hemostasis was checked in the operative field. Cecum, ascending colon and hepatic flexure were mobilized and then laparoscope was removed.
  11. The midline incision was extended over left peri-umbilical area.
  12. The ascending colon was delivered into the incision and points of transection were selected proximally and distally.
  13. Side to side anastomosis was performed with two OpenGIA 80-3.8 mm. Interrupted 3-0 silk Lambert sutures were used to re-inforcement at outer layer
  14. The anastomosis was checked and found to be intact and widely patent.
  15. The small bowel and colon was put back into the abdomen. Pneumoperitoneum was established again.
  16. Possible bleeder was examed again. The orientation of the small bowel was checked thoroughly.
  17. A 12 Fr. penrose drain was placed over Morison pouch and anastomostic site from the RLQ trochar wound.
  18. ADHESION BARRIER FILM was placed below the central abdominal wound.
  19. The fascia was closed with interrupted sutures via Num.1 Vicryl. The fascia of the other trochar wounds were closed with 1-0 Vicryl.
  20. The skin wound was sutured with interrupted method via 3-O Nylon, then covered with Heraderm.
  21. The patient tolerated the procedure well and was brought to the POR in stable condition.
- Estimated blood loss: minimum
- 器械數量: 正確
- 紗布數量: 正確

主任 2062F蔣鋒帆 / 醫師 2336L洪浚嚴

正式摘要(電子簽章：**000000223819941)**

W83 / W83

doctorRank:B
